# Municipal Acute Units as Part of the Clinical Pathway for Older Patients

**DOI:** 10.5334/ijic.4643

**Published:** 2019-10-29

**Authors:** Anne-Kari Johannessen, Sissel Steihaug

**Affiliations:** 1 OsloMet – Oslo Metropolitan University, Department of Nursing and Health Promotion, NO; 2 Health Services Research Unit, Akershus University Hospital, Lørenskog, NO; 3 Ekornveien, NO

**Keywords:** Municipal Acute Unit, clinical pathway, primary care, older patients, qualitative research

## Abstract

**Introduction:**

Since 2016, Norwegian municipalities have been obliged to provide municipal acute 24-hour services representing a service before or instead of hospital treatment. This study explores two municipal acute units (MAUs) as part of the clinical pathway for older patients.

**Methods:**

Patients and healthcare providers from MAUs, purchaser offices, home-based nursing, and physicians were interviewed. Interview transcripts were analysed using systematic text condensation.

**Results:**

The collaboration between the MAU staff and the GPs, the purchaser offices and the home-based services is described as challenging, mostly due to disagreement regarding patients’ admission and discharge. The providers’ different understanding seems to derive especially from where they are working in a way that suits their own work functions.

An exigent collaboration between providers in the MAUs and their collaborative partners hampers the clinical pathway for older patients in the municipal healthcare service.

**Conclusion and discussion:**

When a new healthcare service such as an MAU becomes a part of the clinical pathway in a municipality, it is important to invest a considerable effort in measures designed to strengthen relational and structural collaboration to make the clinical pathway smooth.

## Background

Due to an ageing population and the increasing prevalence of chronic diseases, health authorities in several countries constantly seek ways to improve the quality and efficiency of healthcare services [1,2,3]. In 2012, the Coordination Reform [3] was implemented in Norway to transfer specific responsibilities and resources progressively from the national government to the municipalities. The key aim was to reduce the number of hospital beds, shorten the duration of hospital stays and widen access to healthcare services within the municipality.

Health care in Norway is divided into two broad delivery systems: secondary and primary healthcare systems, each of which is subject to different funding systems, laws and central regulations. Norwegian hospitals are organised within the secondary healthcare system and are a governmental responsibility. They are financed by a combination of block grants and activity-based financing, with hospital employees paid on a fixed-salary basis. The municipalities are responsible for nursing homes, home-based services and social care, which are publicly financed and mainly publicly provided but with an increasing share of private actors. Since 2001, all Norwegian citizens have been assigned to a general practitioner (GP). GPs are paid in part by a capitation component based on their patient caseload and partly on the basis of fee-for-service.

As part of Norwegian Coordination Reform, the establishment of 24-hour municipal acute units (MAUs) constitutes one of several initiatives to improve collaboration and coordination in healthcare services. Since 2016, all Norwegian municipalities have been obliged to have MAU services [3,4,5]. The units are regulated by statutory cooperative agreements between municipalities and regional hospitals. MAUs represent a service before or instead of hospital admission and treatment [3]. The purpose is to prevent hospitalisation and avoid readmissions [4,6]. The official guidelines for MAUs do not specify applicable diagnoses of patient groups and the municipalities are free to organise the service as they see fit. The most common arrangement of MAUs in Norway is in nursing homes, medical centres in relation to a hospital and as municipal or intermunicipal wards [6,7].

### Clinical pathway

Fragmented patient transitions are a well-known phenomenon [8,9,10], especially for fragile and chronically ill elderly patients, and there is a need for better collaboration between providers and between institutions [3,11,12]. Partnership and the sharing of goals and responsibilities are important features of collaboration [13]. Knowledge and understanding of each other’s work, a culture of mutual respect and recognition of each other’s area of expertise and competence, and the free and open exchange of information are also key elements [14]. Interprofessional collaboration can be understood as two or more members of different healthcare professions working together to solve problems or provide services [15]. A clinical pathway is a system for organising collaboration and can be defined as the chronological chain of events that constitutes the patient’s encounter with various parts of the healthcare services with the aim of achieving a continuum of care across settings [3,16,17,18]. The literature uses various concepts to describe the patient’s transition through the healthcare system [16,17]. We have chosen to use the term *clinical pathway* because this focuses on clinical practice and contains certain components of care, treatment and rehabilitation [16,18]. The idea is that clinical pathways (re)organise care by standardising the care process, leading to less variation in care and more transparency on how care is provided [19]. In general, disease-specific pathways increase the length of time between hospital discharge and readmission or death, reduce the total number of rehospitalisations and decrease healthcare costs [20,21]. A review study evaluating the clinical pathway in transition from hospital to primary care shows reduced rehospitalisations and overall high patient satisfaction [22]. A Cochrane review shows that clinical pathways in secondary care settings are associated with reduced in-hospital complications and improved documentation [23]. The feasibility of disease-specific clinical pathways used during hospital discharge and in primary care seems, however, to be limited for patients with complex needs both from a clinical and an organisational perspective [24]. Primary care has to manage the patient’s complex needs holistically, and single-disease guidelines are unsuitable. Røsstad (2013) showed how hospital providers seem to be keen on providing diagnoses, whereas community providers are apparently more concerned with the patient’s functional ability. The development of a patient-centred care pathway across healthcare levels is challenging because of the differing perspectives on care and different organisational structures in secondary and primary care [25]. Care pathways for elderly patients, however, seem to have potential for improving follow-up in primary care by meeting professional and managerial needs for improved quality of care as well as more efficient organisation of home care services [26]. Nevertheless, implementation of this complex intervention in full-time organisations is demanding and requires comprehensive and prolonged efforts at all levels of the organisation (ibid).

Few studies have been conducted in MAUs. One study showed that more than 70% of older patients had experienced problems regarding continuity and transition [27]. Half of the patients studied wanted to be more involved in decisions about their treatment and care, and a quarter of them reported that they were not always treated with respect and dignity. However, one interview study showed that older patients in an MAU particularly appreciate being seen and looked after as a whole human being [28]. The MAU concept is new in the Norwegian healthcare service, and much is being done to establish MAUs in Norwegian municipalities. Hence, knowledge about the importance of MAUs for continuity in the clinical pathway is crucial.

## Objective and context for the study

The objective of the study was to explore the significance of the new concept of the Municipal Acute Unit as part of the clinical pathway for older patients. This study is part of a larger project about Norwegian municipal acute units (MAUs) and collaborating municipalities. The study was conducted in two MAUs in eastern Norway and 8 of 12 municipalities collaborating with the MAUs. MAUs are designed for patients age 18 years and older who need medical observation and treatment for a shorter period (3–5 days). Patients suffering from somatic conditions such as lung infection, diarrhoea, and chronic pain; impaired general condition due to advanced age; and minor mental disorders are the most common [4].

One MAU opened in 2014; it has 16 beds and represents collaboration with 7 municipalities. The other MAU opened in 2016, has 6 beds and represents collaboration with 5 municipalities. The co-operating hospitals’ main obligation is 24-hour telephone guidance on medical issues. The municipalities have reciprocal binding agreements on finances, admission and exclusion criteria. The two units are staffed with nurses and physicians. The physicians do not have night shifts. The MAUs have limited opportunity for diagnostics and advanced treatment. They have an arrangement called the diagnostic loop, which implies that patients are sent to the hospital for rapid diagnostic clarification if the submitting physician is in doubt as to whether the MAU or the hospital can provide the most adequate treatment option. The patients in the two MAUs in 2017 ranged from 18 to 102 years old, with an average of 78 years. In 2017, 71% of the patients from the largest MAU and 49 % from the other MAU were were discharged to their own home [29].

## Method

### Informants

In close collaboration with leaders for the respective workplaces, the first author recruited provider informants from the MAUs, emergency clinics, purchaser offices and home-based care. In addition, 13 physicians representing 8 municipalities were recruited using the snowball method. Healthcare providers from purchaser offices and home-based care as well as physicians were chosen based on who had previously collaborated with MAUs. We aimed to include providers representing both various professional groups and providers on the practical level and leaders. We achieved a reasonable representation in regard to age, gender, position, workplace and municipal affiliation; see Table 1. Of the 40 healthcare providers interviewed, 11 were management-level leaders, and 7 had clinical part-time positions. The first author, who regularly visited the two MAUs over a period of three months, recruited the patients. Patients who were over 50 years of age, able to provide informed consent and were discharged to their homes were asked to participate; 12 agreed to do so. We pursued a strategic selection of informants and achieved a reasonable representation in regard to age, gender, diagnosis and community affiliation of patients; see Table 2.

**Table 1:** Healthcare providers interviewed.

Workplace/profession	No	M	F	Age/Average age	Notes

**Municipal Acute Unit:**				25–64/41	
Nurse	9		9		Respectively 7 from one MAU and 5 from the other MAU involved
Physician	3*	3			
**Purschaser office:**				37–61/47	
Nurse	6		6		Represents 4 municipalities
Social worker	2		2		
Social educator	2	1	1		
**Home-based care:**				22–58/35	
Nurse	8	1	7		Represents 4 municipalities
**Physicians:**				29–67/47	
General practitioner	6**	3	3		Represents 8 municipalities
Medical superintendent	3	3			
Emergency clinic physician	1		1		
Total	40	11	29		

* 1 physician had full time position on MAU, 1 physician had combined position between MAU and Emergency clinic and 1 physician had combined position between MAU, Nursing home and Emergency clinic. ** 4 physicians had full time positions as GP and 2 physicians had combined position as GP and Emergency clinic physician.

**Table 2:** Patients interviewed.

Informant	M	F	Age/Average age	Notes

Patient: 12	5	7	52–90/69	Represents 4 municipalities

### Interviews

Qualitative interviews are suitable when the intention is to explore personal experiences and the meaning people associate with them [30]. The first author conducted semi-structured qualitative interviews by means of interview guides developed in advance. The interviews were based on Kvale’s principles [31]. This meant that the researcher was attentive to the informants’ stories and sensitive to surprises or changes during the interviews that might challenged her preconceptions. The interviews took place from March until December 2017. Each lasted about 45 minutes and was recorded on digital recording equipment and transcribed verbatim by the first author. The patients were interviewed at home one or two weeks after discharge from the MAU. They were asked about their perceptions of admission, their stay, the discharge from the MAU and their first period at home. The providers were interviewed at their workplaces and asked about their work duties, their collaboration with each other and their opinions about the MAU. During the interviews, the emphasis was placed on how informants experienced the collaboration and on the conditions that either promoted or hindered collaboration.

## Analysis

Both authors (a nurse and a physician) analysed the interview transcripts by systematic text condensation as described by Malterud [30]. The analysis was conducted in four steps, alternating among the various steps throughout the process. The first step involved reading all the material to obtain an overall impression and to identify preliminary themes. In step two, we identified meaning units — sections of text representing different aspects of the preliminary themes from the first step — and coded these under different headings, such as “physicians struggled with submitting patients” and “time-consuming collaboration on discharge”. In the coding, we focused on how patients experienced the health services offered and how the providers experienced collaboration and managed collaborations that were challenging. The meaning units were repeatedly sorted into code groups and moved back and forth from one group to another until they were all placed under an appropriate heading. Code groups could be merged or divided, and we ended up with three groups. The meaning units “physicians struggled with submitting patients” and “time-consuming collaboration on discharge” were, for example, gathered under the heading “problematic admission and discharge”. In the third step, we established subgroups exemplifying vital aspects of each code group and analysed each subgroup separately. We then condensed the content of each code group and selected quotations that appropriately illustrated the essence of the descriptions. Finally, we synthesised the condensates from each code group to form a generalised description that reflected the main findings. Each code group was given an appropriate heading. Our example of “problematic admission and discharge” received the final heading of “collaboration on admission and discharge”. The generalised descriptions of the condensates from the three code groups constitute our results and are presented in the results section.

## Ethics

Written informed consent was obtained from the participants before data collection. Participants were informed that their identities and the collected data would be kept confidential, and that they had the opportunity to withdraw from the study at any time. The study followed the Declaration of Helsinki on ethical principles for medical research involving human subjects. The Regional Committee for Medical and Health Research Ethics concluded that the study was not regulated by the Health Research Act (2016/2277/REK sør-øst A). The local privacy protection advisors at Akershus University Hospital HF (ref 17-058) approved the study.

## Results

### Collaboration on admission and discharge

The collaboration between the MAU staff and their collaborating partners — the GPs and the staff at the purchaser offices and the home-based services — was described as partly challenging. Particularly defiant collaboration took place in connection with patients’ admission to and discharge from the MAUs. Providers of different professions and workplaces had different opinions about whether patients were suitable for a stay at the MAUs. MAU employees described a suitable patient as a person with an uncomplicated infection such as cystitis or pneumonia or a person in need of adjusted pain management. However, the GPs and the staff at the purchaser offices and the home-based care nurses argued that this spectrum of patients was too limited, and that other patients also needed institutional beds. All the GPs assessed the criteria as vague and argued that patients with an urgent need for treatment and care were declined at the expense of patients with less complicated conditions. The MAU employees, on the other hand, considered the admission criteria as appropriate and clear.

A majority of the providers were frustrated about how admission criteria, application procedure and the writing of detailed care plans were put into practice by the MAUs, which led to cumbersome collaborations. Most GPs and emergency physicians stated that it was difficult to admit patients to the MAUs because the MAU employees demanded a large amount of patient information, often not readily available in an acute situation at the patient’s home. Several GPs thought that the MAUs had an overly restrictive admission policy. Some GPs described MAU physicians as a little patronising and arrogant when they were contacted. Many GPs preferred to admit patients to the hospital before the MAUs to avoid having to spend an excessive amount of time clarifying and checking out different matters that required diagnostic tools that were not readily available. Some GPs had stopped applying for patient admissions to the MAU.

“Some days ago, I had a home visit to an older physically impaired woman with a fever who seemed to be a little demented. I expected a certain accommodating attitude from my MAU colleague when I requested patient admission. Instead, I got a number of critical and detailed questions to which I struggled to find quick answers. I felt like I was not believed or accounted for, related to the MAU colleague, like a schoolboy in front of his teacher. Moreover, the MAU physician probably had less clinical experience than me”. (GP)

The MAU employees thought that the purchaser offices often made unfair demands as they constantly requested revised care plans when patients were to be discharged. The MAU nurses had to develop detailed care plans to document the patients’ functioning level to the purchasers responsible for establishing possible home-based care. The nurses described the documentation process as difficult and time-consuming and claimed this to be a deliberate tactic to delay the time until patients were received from the MAU. On the other hand, the staff at the purchaser offices described the MAUs’ application procedures for discharge as difficult to follow and unstructured compared to the hospitals’ procedures. Different reporting routines and computer systems in the collaborating municipalities further complicated the collaboration between the MAUs and the home-based services.

Providers regarded the diagnostic loop differently. A few GPs, some of the MAU physicians and all the MAU nurses found the loop necessary to ensure the patient safety, whilst some emergency physicians and GPs considered the loop to be an inappropriate use of time and resources.

“The diagnostic loop works poorly because the hospital emergency room uses an excessively long time to clarify patients. Instead of observation and actions, the patients are often left too long in the waiting zone, unattended and without anything happening. The loop is also a big annoyance for many hospital physicians; they probably have lot of other tasks to perform”. (Emergency physician)

### Conditions within the MAUs promoting or hindering the clinical pathway

In one of the MAUs, several physicians had part-time positions. Both nurses and physicians thought that this weakened the continuity and increased the patients’ stays in the unit.

“After getting an MAU physician in a full-time position, we have achieved a much better procedure system. In addition, systematic drug reviews for each patient and daily whiteboard meetings where the nurses and physicians review and check all the tasks done with each patient have made the management of the unit more rational and safer”. (Nurse)

Almost all nurses felt that night watches were unsafe and stressful because none of the MAUs had physicians present from 22.00–08.00 a.m. During the night, the nurses had only telephonic counselling available, provided by a physician working in the municipal emergency room. This physician was frequently busy treating other patients, and the nurses had to wait even if a situation was assessed as critical. One nurse said:

“I often feel sick to my stomach when night shift is approaching. I even sometimes wonder if I should take sick leave”.

Collaboration in regard to patient discharge between nurses and physicians employed in the MAUs was described as good. Both professional groups said they worked as a team and had trustful communication with one another. One goal for the MAUs is to treat patients suffering from less severe illnesses or conditions and, hence, to lighten the load of the hospitals. According to several nurses employed at the MAUs and the purchasing offices, MAU beds have been used occasionally for older patients in need of more comprehensive care for a longer period, from two up to seven weeks. The MAU physicians argued that, in that way, the MAUs failed their official mission. In addition, they feared that colleagues would resign and look for other jobs. Although the MAU physicians were generally satisfied with their own work situation, some were worried about how the unit had evolved from a treatment and care institution to, more or less, a nursing home.

“Our collaborators put us under constant pressure to fill the beds, and we experience an uncritical use of MAU beds that we must resist. I think, in a way, that they dupe us. Occasionally, we are used as a container for nursing home patients”. (Combined emergency and MAU physician)

The patients were pleased with the kindness and professional competence of the healthcare providers at the MAUs. Notably, they emphasised that the providers’ skills in pain management and developing nutrition plans were very good for helping patients quickly get “back on their feet”. Furthermore, they valued the healthcare staff’s ability to communicate and guide them to regain or improve their health within a reasonable time and to be better prepared to cope with day-to-day life after discharge. Several, however, experienced that narrow patient and shower rooms limited their ability to care for themselves and resulted in patients becoming more dependent on staff assistance. In addition, several patients found it frustrating that they had to relate to many different physicians during their stay and had to repeat their medical history many times. One patient said:

“It was quite strange that I had to relate to six different physicians during the seven days I was admitted, particularly since the unit was so small”.

### The most suitable patients are not necessarily admitted to the MAUs

The patients interviewed received treatment for infections, chronic pain, diarrhoea, dehydration, wounds, malnutrition and general functional failure. All the patients expressed that the treatment they received contributed either to their full recovery or to temporary relief. Patients belonging to the latter group were those with pain problems, wounds and general functional failure. For patients with a need for more comprehensive care, a stay in the nursing home would have been appropriate because expanded home care was insufficient. However, the nursing home was constantly filled; hence, since the MAUs often had available beds, they were regarded as a rescue facility. Some providers categorised this as a misuse of resources. Others thought this was a desperate situation concerning where to put needy patients when the municipality is short of nursing-home beds. Several patients also commented on this lack of institutional beds in connection to MAU discharge. Even though they found the MAU stay to be good, many thought it was too short. They still felt weak and wished they could have further care at another institution.

“The stay was good, but when they said, I had to go home, my spouse and I asked about a short-term stay at the nursing home. Unfortunately, it was full, so I had to go home. Luckily, I got home-based care”. (Patient)

The results show disagreements between providers as to whether the MAUs represent a good service for patients with general functional failure. According to MAU employees, these patients did not benefit from a short stay. They probably needed more time, and the units had no comprehensive treatment to offer. However, several GPs and emergency physicians thought that fragile older patients with age-related ailments and diseases could, nevertheless, benefit from a rest in a peaceful environment that included nutritious food and good nursing.

“The unit just wants uncomplicated, fully diagnosed patients, but they practically do not exist. On the other hand, we have many patients in the 80–90-year age range with functional failure that could benefit from a short period in the MAU. Unfortunately, these patients are almost impossible to get in”. (Combined GP and emergency physician)

## Discussion

### Clinical pathway

The objective of the study was to explore the significance of the concept of the Municipal Acute Unit as a part of the clinical pathway for older patients. For many years, good clinical pathways have been a priority for Norwegian health authorities [3]. The white paper *Living Your Whole Life — A Quality Reform for Elderly* [32] is described as a reform to create a more coherent service for older and chronically ill patients and their relatives, and good patient pathways are recommended as initiatives by which to achieve this. Although a clinical pathway is formulated as a chronological chain of events that constitutes the patient’s encounters with various parts of healthcare services and is interpreted as a way of achieving a continuum of care across settings, this is not an exhaustive definition. We argue that a clinical pathway for older patients should not be precisely defined, except perhaps for patients with specific diagnoses. Patients with similar diagnoses may be very different due to different psychosocial conditions, such as social network and material, mental and cognitive resources. In addition, older people often have a number of diseases and take various medicines and may be frail even without a diagnosis. This implies that developing a clinical pathway must be based on individual conditions. Several collective features must be present in a good clinical pathway. Good collaboration between the providers involved is the crux for developing a streamlined clinical pathway.

### Reluctant collaboration challenges the role of municipal acute units in the clinical pathway

The results show that the providers strived to achieve smooth collaboration, particularly for patients’ admissions and discharges. The MAUs’ requests for detailed patient information from the submitting physician was seen as a hindrance to a streamlined clinical pathway. GPs experienced the admission criteria as vague and the application procedure as time-consuming. GPs have to admit patients with a need for further examination and/or treatment. When they make home visits, they often find themselves in emergency medical situations with limited possibilities to conduct extensive examinations. MAU physicians hesitated to take care of some patients due to their wish to adhere to the units’ guidelines. Several GPs found it difficult to admit patients with comprehensive needs to the units and argued that the MAUs merely wanted patients with uncomplicated medical needs. Older patients suffering from several diseases were refused, even though they probably constitute the majority of the patients in general practice. These patients may, in any case, end up in a hospital. The GPs were frustrated because patients in need of acute care and more comprehensive help were excluded from a stay at the expense of healthier patients. However, another Norwegian study has shown that GPs had positive collaborative experiences with MAU physicians [33]. In addition, nurses in home services claimed that only a narrow sample of patients in the municipality was found to be appropriate for a stay at the MAUs. It is reasonable that MAU nurses’ concerns about the fact that the MAUs have no physician present during the night has an impact on the unit’s admission routines and results in admitting fewer very ill patients.

Nurses in the MAUs and staff at the purchaser offices described their mutual collaboration as somewhat laborious. Employees at the purchaser offices argued that medical documentation in connection to patients’ discharges from MAUs was insufficient, whilst MAU nurses complained about the purchaser offices’ extensive demands for thorough reports requiring several hours of work. Other studies have shown similar results [34,35]. The purchasers assessed the MAU as a respite for the municipal healthcare providers because the nursing home was constantly fully occupied, and the municipalities were often in acute need of institutional beds. In addition, the bustle related to the many tasks in home-based care probably challenged the providers’ collaboration on the clinical pathway. This indicates a deliberate procrastination of time due to a considerable lack of institutional beds and resources available in the municipality.

The challenging collaboration in this study seems to occur across the border between different departments — the MAUs, purchaser offices and primary care. Depending on their workplace, the providers probably interpret the MAUs’ guidelines and functions differently and in a way, that suits their own workplace. The results indicate that different goals, tasks, clinical roles and responsibilities can hinder coordinated clinical pathways. Providers having conflicting responsibilities seem to have problems viewing situations from other providers’ perspectives; this is in line with other studies [34,36,37,38]. According to Christensen et al., different goals can express conflicting interests that might result in tensions between cooperating participants [39]. Decision-making in organisations is often based on constrained rationality. This means that the members of an organisation and those who have the authority to make decisions have a somewhat limited knowledge of the whole picture of the organisation; therefore, they act based on simplified models of reality. This might lead to a selection of certain goals, formulations and means at the expense of others [39]. In this study, the physicians have to admit patients, the MAUs are afraid of getting patients who are too ill and the home-based services are overloaded and want the patients to stay at the MAUs as long as possible. In this way, the departments’ different tasks and goals seemed to impede a smooth clinical pathway.

The professional and interprofessional collaboration in the MAUs seem to work well. Developing relationships and trust between providers requires time and opportunities to get to know each other [14]. The staff in the MAUs worked together daily with the possibility of becoming familiar with each another, and even interprofessional collaboration seemed to function. We argue that collaboration across institutional borders was impeded by time pressures, especially in home-based services, and the lack of places to meet on a face-to-face basis.

### Organisational conditions challenging the role of MAUs in the clinical pathway

Organisational elements also hindered collaboration and impeded a smooth patient flow. The MAU concept constitutes a new part of the existing clinical pathway. It can be perceived as a unit “in between” the hospital and the municipality and is likely to be considered as a new administrative level [40]. This implies increased bureaucratisation due to new procedures and more collaborating partners for submitting physicians and for providers in purchaser offices and home-based services. Nurse leaders are suggested in order to facilitate service development in MAUs [41], but in this study, they do not seem to succeed in creating smooth clinical pathways for older patients.

Informants within the MAUs complained about ineffective treatment programmes. Having several physicians in part-time positions implied that new treatment and care plans often were initiated on the physician’s rounds instead of adhering to the original plan. Many providers considered the diagnostic loop as a hindrance to a smooth pathway. The loop was meant to safeguard patients by providing a second medical opinion from hospital specialists but implied several hours of waiting in the emergency room without any medical assessment. Hence, the result may be uncertainty and, not least, an extra stressful burden for the patient and his/her caregivers.

Several of the patients interviewed described their stay in the MAUs as good but too short and found discharge to be arduous. A limited stay may lead to another institutional stay either at an MAU or a hospital because the disease may flare up again [42]. Such a practice probably does not ease the burden on hospitals.

### Discussion of methods

We used interviews based on interview guides developed in advance to explore how patients and professionals experienced the MAUs. In all, 12 patients and 40 professionals, with different work positions and educational backgrounds, provided varied and multifaceted information. The interviewer is an experienced researcher and has expertise in the field by virtue of her background as a nurse. This made it easy for her to understand what happened in the units but might potentially lead her to disregard or not notice information that a nurse would normally take for granted. The latter was counteracted by her awareness of her own preconception and discussing the findings from the interviews with the co-author, who is a physician. The results were also frequently discussed with research colleagues.

## Conclusion

The study primarily describes how an exigent collaboration between providers in the MAUs, the admitting physicians, the purchaser office and the home-based services hinders the clinical pathway in the municipal healthcare service for older patients. The new MAUs place constraints on the providers’ collaboration. A conscious reorganisation of the Norwegian healthcare services during the past decades, including shorter hospital stays and more patients in primary healthcare, entails a considerably heavier burden on the municipalities [43]. To a limited extent, this increased burden has resulted in more resources being allocated to the sector, not the least nursing homes [44]. When a new healthcare service, such as an MAU, becomes part of the clinical pathway in the municipality, it is important to invest considerable effort on measures designed to strengthen relational and structural collaboration in order to make the clinical pathway smooth.
